# Both Isochronous and Non-Isochronous Metrical Subdivision Afford Precise and Stable Ensemble Entrainment: A Corpus Study of Malian Jembe Drumming

**DOI:** 10.3389/fnins.2016.00285

**Published:** 2016-06-28

**Authors:** Rainer Polak, Justin London, Nori Jacoby

**Affiliations:** ^1^Institute for World Music, Cologne University of Music and DanceCologne, Germany; ^2^Department of Music, Carleton CollegeNorthfield, MN, USA; ^3^Computational Cognitive Science Lab, Department of Psychology, University of California, BerkeleyBerkeley, CA, USA

**Keywords:** rhythmic timing, meter, beat subdivision, ensemble entrainment, audio-based corpus, African drumming, culture

## Abstract

Most approaches to musical rhythm, whether in music theory, music psychology, or musical neuroscience, presume that musical rhythms are based on isochronous (temporally equidistant) beats and/or beat subdivisions. However, rhythms that are based on non-isochronous, or unequal patterns of time are prominent in the music of Southeast Europe, the Near East and Southern Asia, and in the music of Africa and the African diaspora. The present study examines one such style found in contemporary Malian jembe percussion music. A corpus of 15 representative performances of three different pieces (“Manjanin,” “Maraka,” and “Woloso”) containing ~43,000 data points was analyzed. Manjanin and Woloso are characterized by non-isochronous beat subdivisions (a short IOI followed by two longer IOIs), while Maraka subdivisions are quasi-isochronous. Analyses of onsets and asynchronies show no significant differences in timing precision and coordination between the isochronously timed Maraka vs. the non-isochronously timed Woloso performances, though both pieces were slightly less variable than non-isochronous Manjanin. Thus, the precision and stability of rhythm and entrainment in human music does not necessarily depend on metric isochrony, consistent with the hypothesis that isochrony is not a biologically-based constraint on human rhythmic behavior. Rather, it may represent a historically popular option within a variety of culturally contingent options for metric organization.

## Introduction

The rhythms of human music and dance are significantly more complex, more diverse, and more flexible than the rhythmic behaviors found in any other species (see Patel et al., 2005; Bispham, 2006; Fitch, 2006, 2012, 2013; Patel, 2006, 2014; Merker et al., 2009; Bowling et al., 2013; Merchant and Honing, 2014; Ravignani et al., 2014; Merchant et al., 2015). While birds and bonobos may be able to entrain to musical or quasi-musical stimuli exhibiting a constant and acoustically obvious pulse at specific frequencies, adult humans are able to find regular pulses in irregular rhythmic patterns, and at a wider range of tempos, than any other species (McAuley et al., 2006). However, a common presumption in biomusicological studies is that the essence of this human capacity involves the extraction of an isochronous (temporally equidistant) pulse train, which provides a framework for temporal perception and action. Indeed, most approaches to musical rhythm, whether in ethnomusicology (Waterman, 1952; Arom, 1984, 1991; Kubik, 1988, 1994; Tenzer, 2011), music theory (Lerdahl and Jackendoff, 1983; Hasty, 1997; Mirka, 2009), music psychology (Longuet-Higgins and Lee, 1982, 1984; Povel and Essens, 1985; Desain and Honing, 1999; Madison and Merker, 2002), and musical neuroscience (Large and Jones, 1999; Snyder and Large, 2005; Grahn and Brett, 2007; Large, 2008; Grube and Griffiths, 2009; Grube et al., 2010; Nozaradan et al., 2012, 2015; Nozaradan, 2014), as well as biomusicology, presume that human rhythmic entrainment is based on a hierarchical organization of isochronous beats and beat subdivisions. In other words, it is commonplace to regard isochrony as a universal, constitutive feature of the regularity that entrainable rhythms require. Savage et al. (2015) show that isochronous beats represent a statistical universal of near global spread, and the authors suggest that the occurrence of such statistical universals might indicate biological constraints on cultural diversity.

In principle, presuming relative simplicity as a functional prerequisite of metric pulse appears plausible. Together with other mechanisms, such as categorical rhythm perception (Clarke, 1987; Schulze, 1989; Desain and Honing, 2003), it allows one to tell a story of rhythmic evolution along the following lines. While many creatures exhibit isochronous rhythmic behaviors (e.g., locomotive gaits and wing beating, resting respiration, etc.), and while a few can exhibit an isochronous rhythmic response to an external isochronous rhythm (e.g., primate chorusing), humans evolved a capacity for creating endogenous isochronous pulses from more complex stimuli (Merchant and Honing, 2014). Specifically, the relative simplicity of the pulse phenomenon can be understood arising from human behavioral complexity coupled with a need for stable and predictable interpersonal interaction. The temporally predictive functionality of pulse and meter suggests that it should be structurally simpler than the rhythmic structures that give rise to it.

However, this nativist view of a natural predisposition toward isochrony resulting from biological constraints is implausible from a cross-cultural, ethnomusicologically informed perspective. The main thrust of rhythm research in comparative musicology and ethnomusicology has been to emphasize the dramatic range of cultural diversity and difference, not only in their surface rhythms, but also in the metrical systems that function as frameworks for their rhythm perception and production. It is empirically evident that music in many parts of the world makes structural usage of non-isochronous beats, including northern Europe (Kvifte, 2007; Johansson, 2009; Haugen, 2014), south-east Europe (Brăiloiu, 1984; Moelants, 2006; Goldberg, 2015; Polak, 2015), Turkey (Cler, 1997; Bates, 2011; Holzapfel, 2015; Reinhard et al., 2015), Egypt and the Arab world (Marcus, 2001, 2007), Central Asia (During, 1997), India (Clayton, 1997, 2000), and parts of Africa and its diasporas (Gerischer, 2003, 2006; Polak, 2010; Jankowsky, 2013; Haugen and Godøy, 2014; Polak and London, 2014). Both isochronous and non-isochronous beats co-exist in most, if not all, of these regions. Musicians, listeners and respondents —people dancing, singing, working, marching, trancing, or clapping to music—are typically at ease with employing different (yet appropriate) metric frameworks in different pieces of the same repertoires, genres, and styles.

In this paper, performance timings of three pieces of jembe ensemble music from Mali are analyzed to assess whether rhythms characterized by non-isochronous beat subdivisions differ with respect to their precision and stability in complex, polyrhythmic multi-part ensemble music, in comparison to rhythms characterized by isochronous beat subdivisions from the same genre and musical tradition. If isochronous meters are privileged in human rhythm perception and production, then we hypothesize that music that involves non-isochronous beat subdivisions should exhibit less precision and stability than music with isochronous beat subdivisions. In particular, we would expect rhythms produced in a non-isochronous context to display:

- Greater variability in the onset timing (i.e., micro-rhythmic placement of drum strokes within the metric cycle);
- Greater asynchrony amongst members of the ensemble (i.e., micro-rhythmic deviation of note onsets by two or more players in the same metric location).

Within a corpus of isochronous and non-isochronous pieces displaying otherwise similar characteristics, the aforementioned hypothesis predicts that non-isochronous pieces will display greater timing variability and greater ensemble asynchrony in comparison with isochronous pieces. If, however, we find that variability and asynchrony of non-isochronous rhythms are not substantially different than isochronous rhythms, then one can no longer claim that isochronous meter has a privileged status in human rhythm perception and production.

## Materials and methods

### Music and recordings used in this study

The music we have studied is colloquially known as “jembe music,” as the jembe (also djembe) is featured as main instrument in these ensembles. The jembe is a goblet shaped drum beaten with bare hands, originating from Guinea and Mali. Traditionally, jembe-centered percussion ensemble music has played a central role in celebratory dance events such as weddings and other life cycle events, as well as with agricultural work-tasks such as hoeing fields for weeding. In the 1960s, jembe music and dance entered programs of state-sponsored folkloric ensembles and, at the same time, became part of the urban popular culture in Bamako, Conakry, Dakar, and Abidjan, among other West African cities. Since the 1980s, West African jembe music, musicians, and instruments have migrated globally (see Charry, 1996, 2000, chapter 4; Polak, 2000, 2004, 2005, 2007, 2012). The popular, vernacular, and participatory characteristics of jembe music make it a particularly relevant case for issues in the psychology and biology of music, because these qualities, which are typical of many types of functional music, are arguably more representative of human musicality than, for instance, Western art music (Peretz, 2006).

Malian drum ensembles typically involve three distinct musical roles: a variative lead drum, a repertoire-specific timeline, and one or more ostinato accompaniment parts (Polak and London, 2014). These roles are assigned to specific instrumental “voices” or ensemble parts. In the Bamako style of jembe music performance in the 1990s and early 2000s, the minimum ensemble size was a duet of one jembe playing the lead part and one dundun, a cylindrical drum beaten with a stick, playing the timeline. Trios add a second jembe playing an ostinato accompaniment rhythm; if financial and logistic resources allow for it, a second dundun is added to further support the accompaniment section.

The set of recordings analyzed here is comprised of three different pieces: Maraka, Manjanin, and Woloso. These three are among the core repertoire of standard pieces in the Bamako style of jembe music (see Polak, 2012). The pieces in our corpus involve two different meters, and were performed by three different ensemble sizes and with four different lead drummers (see Table 1).

**Table 1:** **Set of recordings**.

**Recording**	**Player Jembe 1**	**Player Dundun 1**	**Player Jembe 2**	**Player Dundun 2**	**Ensemble size**	**Playing time**	**Beat IOI (ms) start of piece**	**Beat IOI (ms) end of piece**
Maraka-1	D. Kone	M. Jakite	S. Balo		trio	3:00	472	328
Maraka-2	D. Kone	M. Jakite	S. Balo		trio	2:15	528	336
Maraka-3	S. Balo	M. Jakite	D. Kone		trio	2:30	582	341
Maraka-4	J.M. Kuyate	M. Jakite	D. Kone		trio	2:15	536	306
Maraka-5	J.M. Kuyate	M. Jakite			duet	2:00	549	306
Maraka-6	I. Coulibaly	M. Jakite	D. Kone	A. Traole	quartet	4:50	566	333
Manjanin-1	D. Kone	M. Jakite	S. Balo	A. Traole	quartet	5:00	444	295
Manjanin-2	D. Kone	M. Jakite	D. Kone		duet	4:20	523	305
Manjanin-3	S. Balo	M. Jakite			trio	3:15	443	304
Manjanin-4	I. Coulibaly	M. Jakite	D. Kone	A. Traole	quartet	5:40	500	307
Woloso-1	D. Kone	M. Jakite	S. Balo		trio	3:45	588	381
Woloso-2	D. Kone	M. Jakite	S. Balo	A. Traole	quartet	3:20	499	319
Woloso-3	D. Kone	M. Jakite			duet	3:10	597	333
Woloso-4	S. Balo	M. Jakite	D. Kone		trio	2:40	502	339
Woloso-5	J.M. Kuyate	M. Jakite	D. Kone		trio	2:00	535	322

As is typical of jembe music performance, all recordings show a large-scale, nearly continuous structural accelerando; the tempo at the end of each piece is 30–45% faster than in the beginning. The average ending tempo of 185 bpm (IOI = 324 ms per beat) is very rapid, yielding an average IOI of 108 ms per metric subdivision, which is near the limit for sensori-motor synchronization (Repp, 2003). Their rhythmic textures are near-maximally saturated, that is, each time-point at the subdivision level almost always receives a note onset. Typically, no single player articulates every time-point in the metric cycle for more than a few cycles. Rather, the saturated rhythmic texture results from the interweaving phrases of various ensemble members playing together.

**Figure 1:**
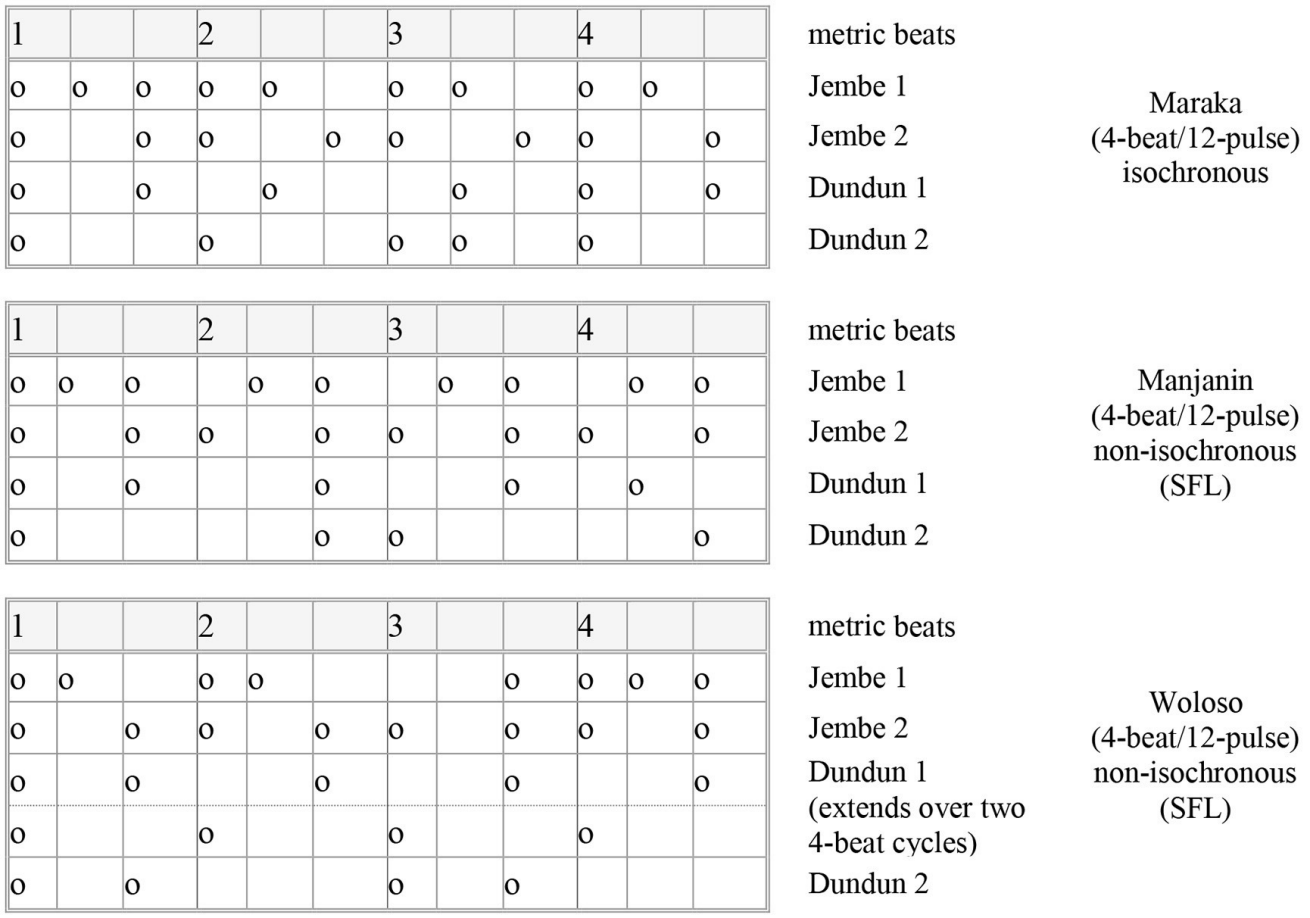
**Rhythmic patterns (melodic and timbral aspects omitted) for Maraka, Manjanin, and Woloso in annotated box notation**. The pattern given for Jembe 1 is an example of a typical lead drum phrase.

The three studied pieces share a common type of metric framework: a cycle of four regular beats with ternary subdivision. Polak (2010) found two different timing patterns for the ternary subdivision timing in these three pieces. Maraka has quasi-isochronous triplets, while the non-isochronous or “swung” ternary subdivision in Manjanin and Woloso consistently showed either a short-medium-long (SML) or short-long-long (SLL) pattern, which were assumed to represent variations of a slightly more generic pattern type, short-flexible-long (SFL). These patterns appeared stable for each piece, across different recordings, players, ensemble parts, durations, phrases, and tempo changes, and thus seemed to represent repertoire-specific metric norms. They were found in other types of drum ensemble music from Mali as well (Polak and London, 2014). Figure 1 graphically represents the basic drumstroke patterns used by each part in each piece. Note that the column widths are indicative of their characteristic timings.

### Data collection and preparation

In 2006/07, author RP collected a set of 15 multi-track audio and video recordings of complete live drum performances while conducting ethnographic field research in Bamako, Mali. Unidirectional microphones (AKG C-419) were clipped-on to the rims of each drum. Individual parts were recorded to a mobile digital four-track studio (Edirol R4) in WAVE-file format at 16-bit/48-Hz. A mini-DV camcorder (Canon XM2) captured video footage at 25 progressive scans per second. Recording sessions took place in the open air, where there was little acoustical crosstalk of instruments and reverberation from walls. The single tracks of the multitrack recordings proved clean enough for audio analysis without the need for frequency filtering.

Audio and video recordings were combined and synchronized in Vegas Pro 11 and 12 (Sony); Soundforge Pro 10 (Sony), Wavelab 7 (Steinberg), and Cubase 7 (Steinberg) were used for onset detection and marking. Onsets were detected automatically, and then were individually checked by eye. Note onset times were exported to Excel 2013 for data organization, and then to Matlab 8 (Mathworks) for further analysis. Out of the 42,297 resulting onsets, some 1054 data points (2.5% of all onsets) were excised from the beginning and end of recordings, to exclude informal introductions and formulaic endings that do not conform to the stable polyrhythms of interest in our study.

Given the structural tempo changes in each recording, analyzing timing data as absolute durations (in seconds or milliseconds) would be disadvantageous, because the magnitudes of resulting values then would be incomparable across the greatly different tempos covered in the performances. We therefore chose the four-beat metric cycle as the basic unit of analysis and normalized (“detrended”) the time-series from the tempo factor by giving temporal intervals as percentages of the local four-beat cycles. To obtain this, we performed the following process:

1. The beginning of each four beat cycle was identified using the ostinato accompaniment of Jembe 2 or, in case of duets where Jembe 2 is not present, of the timeline phrase of Dundun 1.
2. We identified all onsets in all instruments within a tight window around the start of the Jembe 2 cycle.
3. We computed the average of the identified onsets, and marked it as the beginning of the four-beat cycle.
4. Other onsets within the piece were normalized to the relative position between two adjacent cycle starts.

All normalization was done only at the four-beat cycle level; we did not normalize each beat independently.

**Figure 2:**
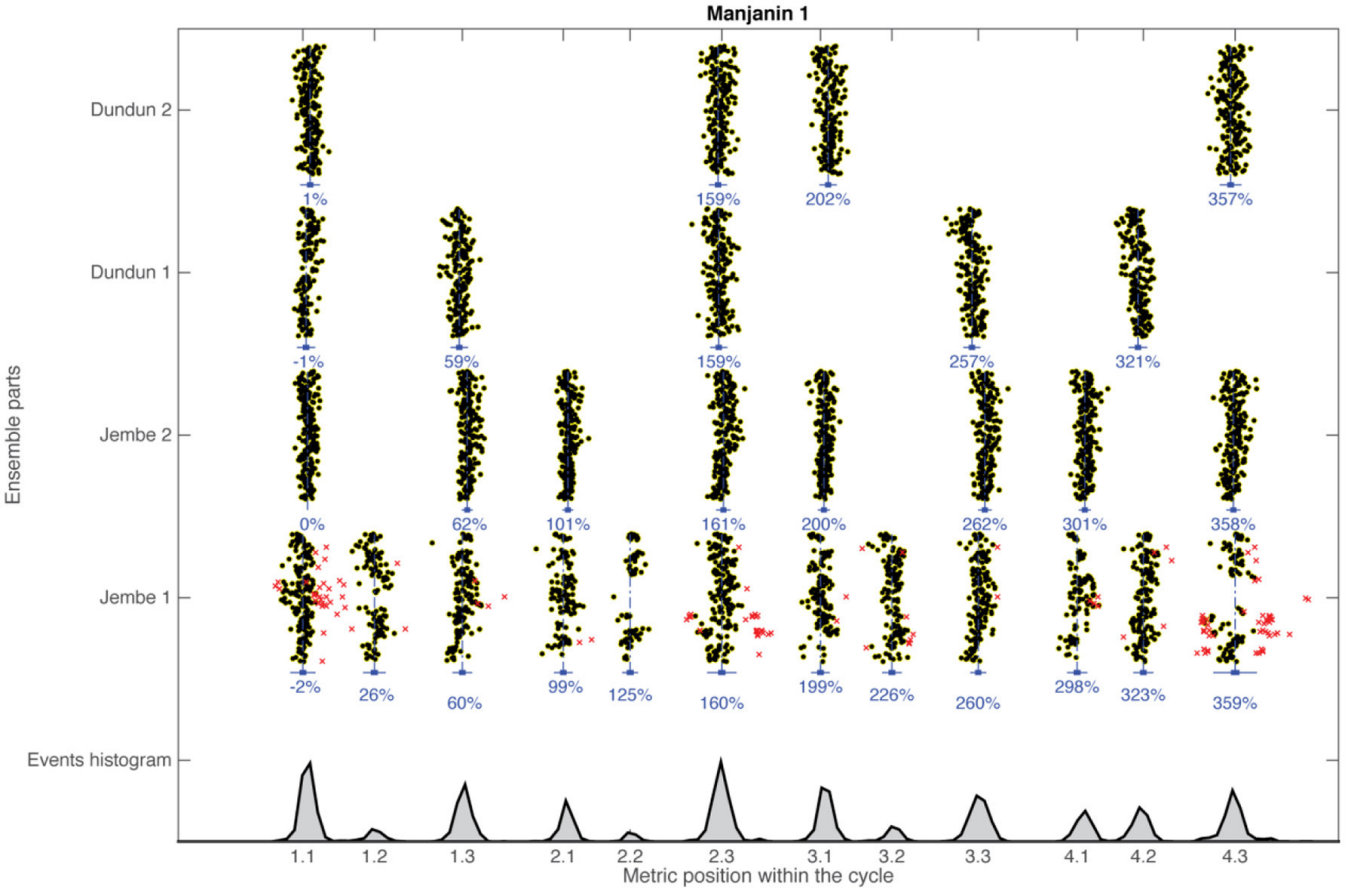
**Top** all 5393 onsets of four ensemble parts aligned to the average downbeat (Pulse 1.1) in a sample recording (Manjanin 1). The distribution of onsets within the metric cycle is plotted on the x-axis; the course of absolute time (sequence of metric cycles) is plotted on the y-axis for each ensemble part. Numbers below each vertical extension give the mean distributions of onsets for each metric position relative to the four-beat cycle (= 400%). Ornamental filtered events are marked with red crosses. **Bottom** shows the histogram of all events included in the piece. Histogram peaks correspond to the locations of metric subdivisions.

**Figure 3:**
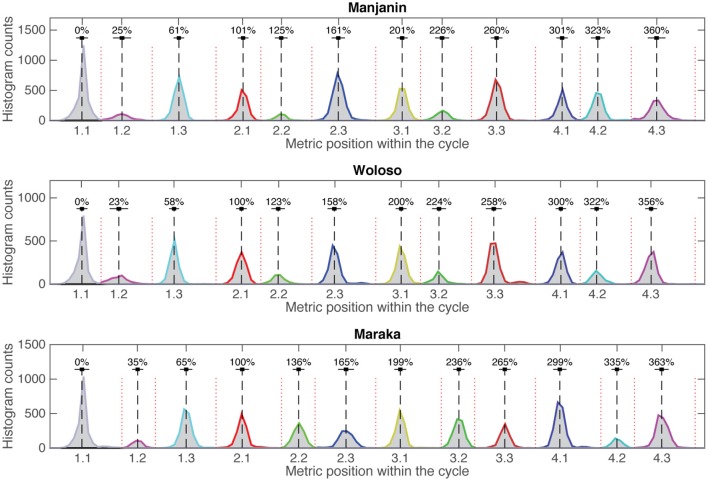
**Aggregated onset histograms per piece (***n*** = 41,243)**. Red dotted vertical lines specify the heuristically defined location of metric pulse-bin borders. Numbers above dashed black lines show the mean location of the onset within a pulse-bin relative to the four-beat cycle (= 400%).

Figure 2 (top) shows the result of this process for one piece in the corpus. Despite the large tempo changes (in this piece from 136 bpm to 197 bpm) the onsets are organized in a highly structured fashion. Figure 2 (bottom) shows the aggregated histogram of all onsets, each peak corresponding to one of the 12 metrical grid positions. Figure 3 shows that these peaks were also consistent across renditions of the same piece. The strictness of adherence to the metric grid for each piece is striking, justifying the heuristic for the identification of the cycle start. Based on this structure we also defined heuristic boundaries between metric positions (displayed in red in Figure 3) and binned each onset to the corresponding metric bin. The exact location of the boundaries does not matter much for the binning process, as the peaks are extremely well separated. However, a small percentage (less than 3%) of all events was nevertheless positioned in ambiguous locations near the heuristic boundaries. These events almost exclusively represent metrically extraneous onsets by the lead-drum part. The first jembe frequently embellishes phrases by adding extra ornamental strokes. These include flams, which consist of two onsets that perceptually merge into one rhythmic event, as well as rolls that combine three or more strokes at a frequency higher than that of the metric subdivision. The approach to filtering these extraneous onsets was two-fold. First, we assumed that only one event within each subdivision “bin” would function as the articulation of that particular subdivision pulse and hence be relevant for ensemble synchrony. Whenever one metric pulse-bin received two onsets by the lead drum, we discounted the onset that was more distant from the mean value for that metric position. Secondly, we defined windows of 17% of the normalized beat duration for each of the three subdivisions (that is, about half of their nominal normalized duration), spread asymmetrically (−10% to +7%) around the mean value for each of them, and discarded all onsets outside that window. Author RP, an expert in this style of music, verified that the decision made by this heuristic corresponded to his understanding of the musical style by visual and audio inspection of the entire corpus. In any case, the number of filtered events was small, totaling merely 1170 events (2.8%) of all events in the corpus.

## Results

### Isochronous vs. non-isochronous subdivision timings

**Figure 4:**
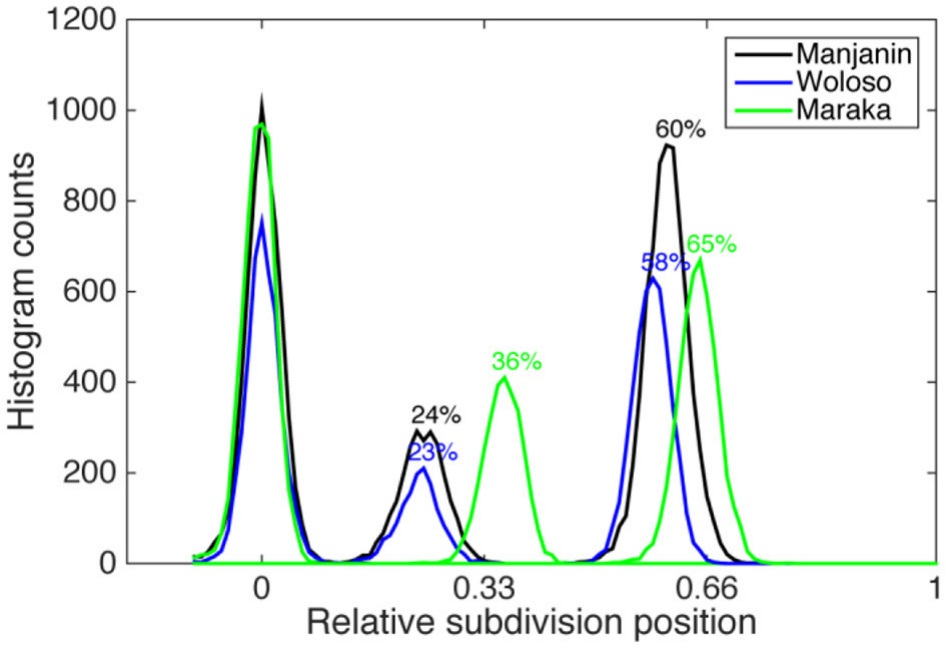
**Onset histogram of all events of all ensemble parts and all recordings relative to the normalized local beat duration (1 beat = 100 %) for the three pieces in the corpus**.

**Figure 5:**
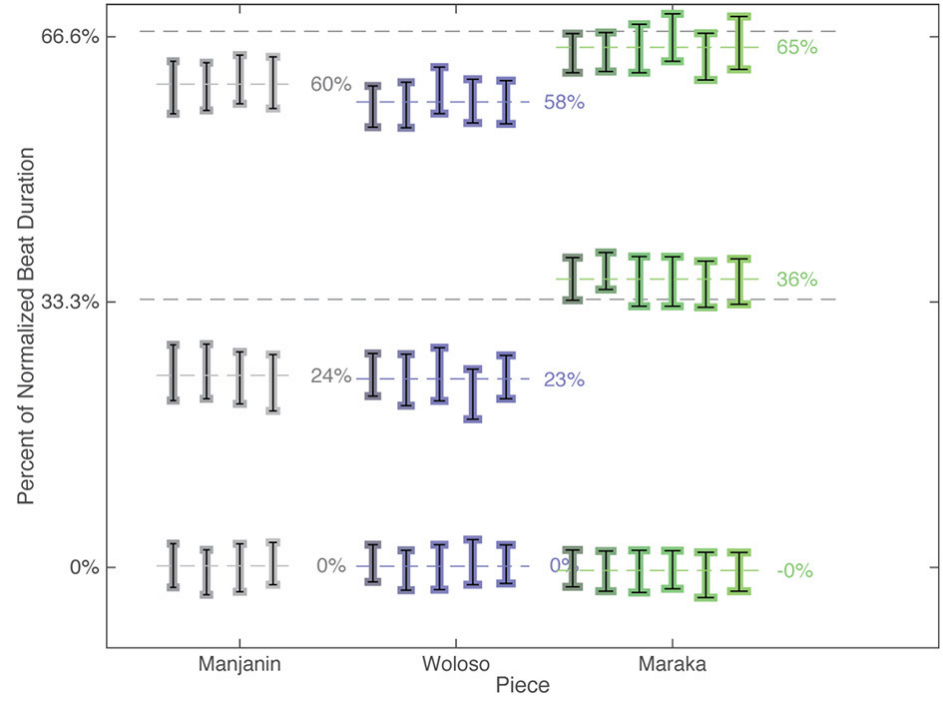
**Mean subdivision timing ratio grouped by piece and recording**. Error bars represent the standard deviation of the subdivision position (1 beat = 100%) computed for each recording individually. Dashed lines represent idealized isochronous subdivisions.

All three pieces exhibit a meter comprised of four isochronous beats that show almost no local differences in IOI. However, within each beat, the three pieces show two distinct patterns of subdivision timing (see Figures 4, 5). The difference is particularly evident in the second (middle) subdivision pulse-bin. In Maraka the subdivisions are nearly isochronous, albeit with a characteristic slight compression of the middle element (see Desain and Honing, 2003; Repp, 2005; Repp and Su, 2013). By contrast, Manjanin and Woloso display a short-medium-long pattern of subdivision, with an earlier articulation of the middle element.

**Figure 6:**
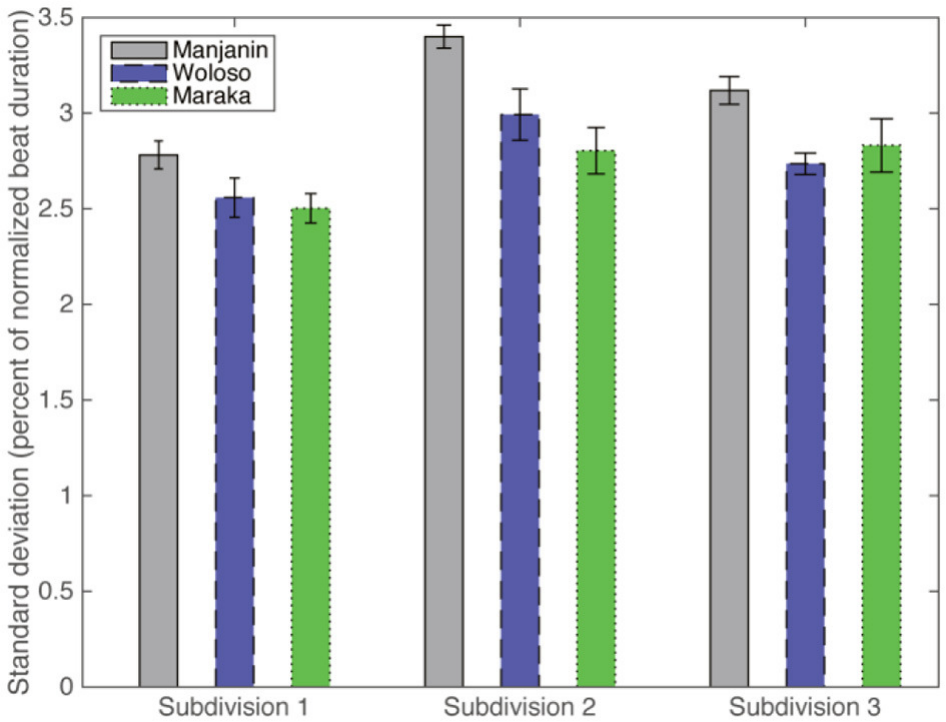
**Standard deviation of subdivision durations, all ensemble parts, all recordings, as percentages of the normalized local beat duration, separated by piece**. Error bars represent standard error of the mean.

As can be seen in Figure 6, the variability of subdivision timing is very low on average; the standard deviations of all onsets in all recordings for each of the three pulse classes are approximately 2.5–3.5% of the local beat duration.

We further analyzed these variabilities with a 2-way Piece × Subdivision ANOVA that shows both a significant main effect of Piece [*F*(2, 36) = 10.7, *p* < 0.001 and of Subdivision *F*(2, 36) = 13.6, *p* < 0.001], but no significant interaction [*F*(4, 36) = 0.96, *p* = n.s]. *Post-hoc* tests showed that (a) there is no significant difference in variability between the isochronous Maraka and the non-isochronous Woloso [*t*(31) = 0.47, *p* = n.s], whereas the variability of Manjanin was significantly larger than both Woloso [*t*(25) = 3.05, *p* = 0.016] and Maraka [*t*(28) = 3.46, *p* = 0.005] (Bonferroni correction for multiple comparisons applied here and in all post-hoc tests noted below); (b) the variability of the first subdivision (onbeat) is significantly smaller than both the second subdivision [mid-beat; *t*(25) = 3.04, *p* = 0.016], and the third [up-beat; *t*(28) = 3.45 *p* = 0.005], which were not significantly different from one another [*t*(31) = 0.47, *p* = n.s]. This is consistent with the idea that the strong metric positions (onbeat) are more stable than weak metric positions (London, 2012; see Repp, 2003 for similar result in a finger tapping experiment).

To test the consistency of variability over the large tempo changes within each performance, we divided each recording into two parts with the same number of four-beat cycles. The average tempo of the second half of the pieces (168 BPM) was significantly faster than the beginning half [145 BPM; *t*(14) = 16.3, *p* < 0.001]. However, the differences between the first and second half in terms of relative performance variability were extremely small: 2.7 and 2.9%, respectively. While it is to be expected that relative variability will increase with tempo (Wing and Kristofferson, 1973), a 3-way Piece × Subdivision × Part (first vs. second half) ANOVA showed only a marginally significant main effect of Part [*F*(1, 76) = 4.07, *p* = 0.05] but a significant Part × Piece interaction [*F*(2, 76) = 3.53, *p* = 0.03]. However, a *post-hoc* test only found a significant contrast between the end of the Manjanin pieces and all other possible parts and pieces (*p* < 0.05) (Bonferroni correction for multiple comparisons applied here and in subsequent *post-hoc* tests). Importantly, there was no statistically significant difference between isochronous Maraka and non-isochronous Woloso among all the possible tested situations, i.e., the beginning and ending of the piece and each of the three possible subdivisions (*p* ≤ 0.05). These results show that (a) the basic subdivision timings (Figures 2, 3) are highly stable in all three pieces, and (b) there is no significant difference in variability between the isochronous Maraka and the non-isochronous Woloso.

### Asynchronies between ensemble parts

**Figure 7:**
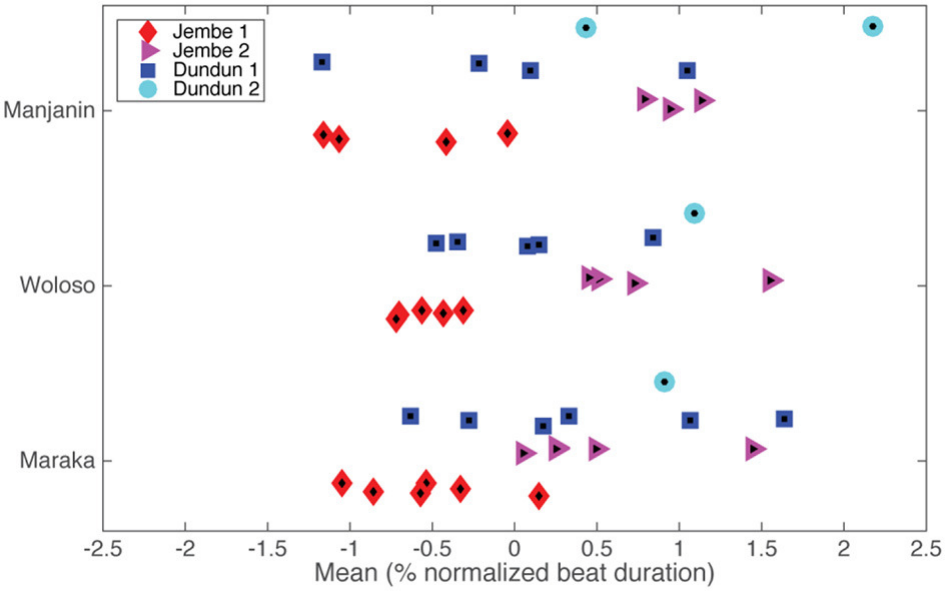
**Mean signed asynchronies between ensemble parts, grouped by piece and instrument**. The asynchronies are given relative to a virtual reference beat (zero asynchrony) calculated as the mean of all onsets (per recording) present in a metric position. Colors and shapes represent individual instruments within a piece. To improve visibility of almost overlapping values some random jitter was added to the y-axis of this graph.

To assess the precision of coordination among parts and to provide a window on the performers' use of a common metric framework, we measured the extent, pattern, and variability of the mean asynchronies between onsets by different individual ensemble members in the same metric position. Mean signed asynchronies were calculated relative to a virtual reference beat, which we defined as the mean of all onsets within each metric bin for each performance. Across all three pieces in the corpus, the value of the mean signed asynchronies is about 2% of the normalized local beat duration (see Figure 7). Depending on the tempo (beat IOIs from ≈300 to ≈600 ms), these mean asynchronies are in the range of 6–12 ms.

A 2-way Piece × Instrument ANOVA shows a significant main effect of instrument [*F*(3, 34) = 14.1, *p* < 0.001] but no significant effect of piece [*F*(2, 34) = 0.01, *p* = n.s] nor significant interaction [*F*(6, 34) = 0.52, *p* = n.s]. *Post-hoc* tests found that the lead drummer (Jembe 1) tended to play ahead of the accompanists [Jembe 2: *t*(25) = 7.92, *p* < 0.001; Dundun 2: *t*(17) = 6.7, *p* < 0.001] as well as ahead of the timeline [Dundun 1: *t*(28) = 3.39 *p* = 0.012]. Another related measure of accuracy is the absolute value of the mean asynchrony: a 2-way Piece × Instrument ANOVA did not show any significant main effect [piece: *F*(2, 34) = 0.59, *p* = 0.55; instrument: *F*(3, 34) = 1.47, *p* = 0.24] nor an interaction [*F*(6, 34) = 0.42, *p* = 0.85]. Taken together, these results show that the pattern and extent of asynchrony between players does not vary between pieces; isochronous and non-isochronous pieces do not differ in this respect.

**Figure 8:**
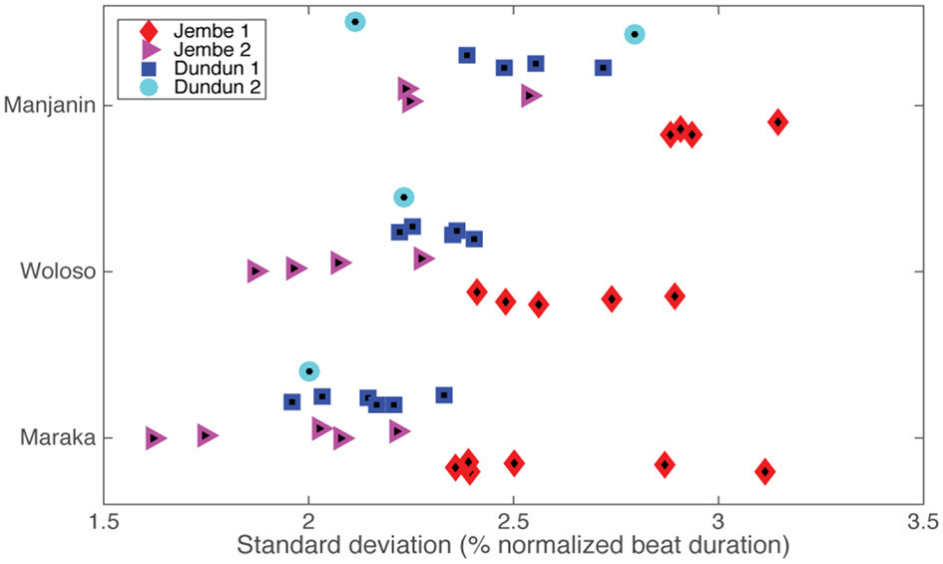
**Standard deviation of the asynchrony, grouped by piece and instrument**. Colors and shapes represent individual instruments within a piece.

The variability of asynchronies is also low (standard deviations range between 1.5–3.2% of the local beat duration), indicating that the minimal amount of mean asynchrony does not result from averaging out larger deviations, but represents a very stable pattern of highly precise ensemble timing (see Figure 8)^1^.

Analyzing the standard deviation of the asynchronies with 2-way Piece × Instrument ANOVA showed significant main effect of piece [*F*(2, 34) = 13.2, *p* < 0.001] and instrument [*F*(3, 34) = 21.9, *p* < 0.001] but no significant interaction [*F*(6, 34) = 0.19, *p* = 0.97]. *Post-hoc* analyses show that the isochronous Maraka and non-isochronous Woloso do not significantly differ from each other [*t*(31) = 0.96, *p* = n.s], but are significantly less variable than the non-isochronous Manjanin [*t*(29) = 3.05, *p* = 0.005]. In addition, the *post-hoc* analysis showed that Jembe 1 has a significantly larger variability compared with Jembe 2 [*t*(25) = 6.12, *p* < 0.001] and Dundun 1 [*t*(28) = 4.57, *p* < 0.001]. However Jembe 1 and Dundun 2 were not significantly different from one another [*t*(17) = 2.55, *p* = n.s]. Note, however, that all these differences and nominal values are extremely small. For example, the differences are less than 1% of the beat duration, and the largest nominal value of variability (Jembe 1: 3.3%) represents a timing difference of only 10–20 ms.

## Discussion and conclusion

This paper examines the assumption that isochrony is privileged in human rhythmic perception and production by testing the hypothesis that the production of non-isochronous rhythms will be associated with both greater durational variability as well as larger and less stable inter-personal asynchronies in ensemble performance. We analyzed three pieces whose rhythms are characterized by either isochronous or non-isochronous meters. Manjanin and Woloso share a similar short-flexible-long subdivision timing pattern that is different from the quasi-isochronous subdivisions in Maraka (Figures 4, 5). This hypothesis predicts much smaller and less variable asynchronies among ensemble members performing the isochronous Maraka than in performances of both non-isochronous Woloso and Manjanin. However, our results are inconsistent with this prediction in three main ways:

1. We found that the average extent and variability of asynchronies in all three pieces was extremely small (less than 3% of the beat duration) and relatively stable as the piece progresses (the mean change between first and second half of the pieces was only about 0.2%). The extent of asynchronies in the jembe ensemble (6–12 ms) is considerably smaller than in European art music and African-American jazz ensemble performances, where the lower end of typical ranges lies at 20–30 ms (Rasch, 1979; Shaffer, 1984; Rose, 1989; Prögler, 1995; Friberg and Sundström, 2002; Goebl and Palmer, 2009; Timmers et al., 2014). Jembe drummers in both isochronous and non-isochronous contexts remain very tightly synchronized to each other and they do so with cutting precision and rock-solid stability (Figures 2, 7, 8).
2. The extent of asynchrony among the ensemble members—the mean signed asynchronies as a percentage of the normalized beat duration (see Figure 7)—was *not* significantly different among the three pieces.
3. The variability of the relative position and asynchronies of onsets—indicative of the relative stability of entrainment among ensemble members—showed significant and consistent differences between the pieces, but these differences did not follow the isochrony hypothesis: isochronous Maraka and non-isochronous Woloso were not significantly different. The non-isochronous piece Manjanin had a small but significant increase in variability compared with both non-isochronous Woloso and isochronous Maraka.

While music based on isochronous pulses is held to represent a statistical universal (Savage et al., 2015), it remains that (a) music based on non-isochronous pulse structures is found in many cultures (referenced in the introduction) and (b) non-isochronous pulse structures afford precise and stable rhythmic performance and entrainment, as our study above has shown. This forces one to conclude that isochrony is not an inherent, biologically-based constraint on human rhythmic behavior. Rather, it may represent a historically popular option within a variety of culturally contingent options for metric organization. A range of evidence supports this assumption. First, Hannon and colleagues have demonstrated in a series of experimental studies that enculturation overrides the mathematical complexity inherent in non-isochronous beats. Non-isochronous beat sequences such as 2+2+3 are more difficult than isochronous ones for Western adult listeners, but *not* for Bulgarian, Macedonian, Turkish, and Indian listeners (Hannon and Trehub, 2005a; Hannon, 2010; Hannon et al., 2012a; Kalender et al., 2013; Ullal-Gupta et al., 2014). Studies of rhythmic development have shown that 6-month-old infants can respond to isochronous and non-isochronous beats with equal facility, but by 12 months, infants already develop a bias toward the rhythms of their environment. Yet one-year-old infants can quickly learn to adapt to “foreign” (e.g., non-isochronous) rhythmic patterns through brief exposure (Hannon and Trehub, 2005a,b). Statistical learning by passive exposure quickly and strongly shapes our perception and cognition of rhythm and meter (Hannon et al., 2012b). The transition from culture-general to culture-specific patterns in beat perception starts very early in life, and the privileging of isochronous over non-isochronous beats is on the culture-specific, not on the culture-general side of the developmental divide (Hannon and Trehub, 2005b).

Second, long-term ethnographic research in Malian jembe music (author RP) reveals that local players, listeners, and dancers do not experience non-isochronous subdivisions as relatively difficult or irregular, nor do they conceptually distinguish them from isochronous patterns. For instance, professional teachers do not try to avoid non-isochrony when students show difficulties in understanding a rhythm.

Biomusical discussions of the nature of human rhythmic and entrainment capacities emphasize the diversity and flexibility of human rhythmicity, while at the same time presuming that these complex behaviors supervene upon a small number of simple underlying metrical processes. However, from our study and the other cross-cultural studies of rhythm cited above, it is evident that the human capacity for rhythm, and pulse perception and production in particular, may be more complex than previously assumed. Metric flexibility is surely limited in degree when compared to rhythmic flexibility, yet clearly metric regularity does not depend upon isochrony, though this has been supposed in many theoretical, analytical, and psychological accounts of rhythm in Western classical and popular music.

This re-characterization of the human capacity for rhythm and entrainment further emphasizes the distinction of humans from all other species. For example, fireflies have one meter/rhythm (without rhythm-meter distinction), whereas birds and great apes may have a few rhythms and one meter, within narrow limits of tempo (Schachner et al., 2009; Patel et al., 2009a,b; Patel, 2014; Ravignani et al., 2014; Large and Gray, 2015). Humans, by contrast, are able to perform a great many rhythms at many different tempos; contrary to conventional presumptions, they also perceive many more meters than time signatures in Western musical notations suggest (London, 2012). Humans are able to adapt to a much broader range of rhythmic situations and contexts partly because their capacity for meter, too, is more flexible and differentiated. One aspect of the flexibility and source for differentiation of meters is that metric pulses do not need to be isochronous—neither their beats nor their subdivisions.

Biomusicological studies of rhythm hotly contest the rhythmic abilities of non-human animals. By contrast, they seem to assume that our understanding of the human capacity for rhythm and entrainment is more or less fully understood, or at least fully documented. This is premature. In particular, existing and emerging knowledge about cultural diversity has not been sufficiently integrated into music theoretic, psychological, neuroscientific, and biological discussions of human rhythmicity (for recent, surprisingly innovative insights of such perspective in other domains such as economic behavior, visual perception, or spatial cognition, see Henrich et al., 2010a,b). This bears the risk of distortion, since the standard contexts for the evolution, history, and practice of human music and dance are marked by the *encultured* development of individuals and the *encultured* social situations and institutions of individual action and social interaction. The definition of the human capacity for rhythm needs to recognize that cultural diversity and flexibility are part and parcel of human nature.

## Author contributions

All authors contributed equally to the paper. Author RP collected the data. Authors RP, JL, and NJ analyzed the data and wrote the paper.

## Funding

Data collection was funded by Deutsche Forschungsgemeinschaft (DFG), research grant PO 627/3-1.

### Conflict of interest statement

The authors declare that the research was conducted in the absence of any commercial or financial relationships that could be construed as a potential conflict of interest.

## Footnotes

^1^Note that the standard deviations of the asynchronies were computed similarly to the mean signed asynchrony: separately for each piece, metric position, and instrument. This computation is therefore slightly different from the standard deviation computed in Figure 6, in which onsets within different metric bins (in the four-beat cycle) that are associated with the same metric subdivision (onbeat, midbeat, or upbeat) were aggregated independently of whether referring to Beat 1, 2, 3, or 4 in the four-beat cycle. Note that both methods provide consistent results (compare Figures 6, 8).

## References

[B1] Arom S. (1984). Structuration du temps dans les musiques d'Afrique Centrale. Revue Musicol 70, 5–36. 10.2307/928652

[B2] Arom S. (1991). African Polyphony and Polyrhythm. Musical Structure and Methodology. Cambridge; New York, NY; Paris: Cambridge University Press.

[B3] Bates E. (2011). Music in Turkey: Experiencing Music, Expressing Culture. Oxford: Oxford University Press.

[B4] Bispham J. (2006). Rhythm in music: what is it? Who has it? And why? Music Percept. 24, 125–134. 10.1525/mp.2006.24.2.125

[B5] Bowling D. L. Herbst C. T. Fitch W. T. (2013). Social origins of rhythm? Synchrony and temporal regularity in human vocalization. PLoS ONE 8:e80402. 10.1371/journal.pone.0080402 24312214 PMC3843660

[B6] Brăiloiu C. (1984). Aksak rhythm, in Problems of Ethnomusicology, ed Brăiloiu C. (Cambridge: Cambridge University Press), 133–67.

[B7] Charry E. (1996). A Guide to the Jembe. Percus. Notes 34, 66–72.

[B8] Charry E. (2000). Mande Music: Traditional and Modern Music of the Maninka and Mandinka of Western Africa. Chicago, IL: University of Chicago Press.

[B9] Clarke E. F. (1987). Categorical rhythm perception: an ecological perspective, in Action and Perception in Rhythm and Music, ed Gabrielsson A. (Stockholm: Royal Swedish Academy of Music), 19–34.

[B10] Clayton M. (1997). Le metre et le tal dans la musique de l'Inde du Nord. Cahiers Musiques Traditionnelles 10, 169–189. 10.2307/40240271

[B11] Clayton M. (2000). Time in Indian Music. Rhythm, Metre, and Form in North Indian Rag Performance. Oxford: Oxford University Press.

[B12] Cler J. (1997). Aksak: les catastrophes d'un modèle, in Cahiers de Musiques Traditionnelles, Vol. 10, 60–80. Available online at: http://ethnomusicologie.revues.org/831

[B13] Desain P. Honing H. J. (1999). Computational models of beat induction: the rule-based approach. J. New Music Res. 28, 29–42. 10.1076/jnmr.28.1.29.3123

[B14] Desain P. W. M. Honing H. J. (2003). The formation of rhythmic categories and metric priming. Perception 32, 341–366. 10.1068/p3370 12729384

[B15] During J. (1997). Rythmes ovoïdes et quadrature du cycle. Cahiers Musiques Traditionnelles 10, 17–36.

[B16] Fitch W. T. (2006). The biology and evolution of music: a comparative perspective. Cognition 100, 173–215. 10.1016/j.cognition.2005.11.009 16412411

[B17] Fitch W. T. (2012). The biology and evolution of rhythm: unraveling a paradox, in Language and Music as Cognitive Systems, eds Rebuschat P. Rohrmeier M. Hawkins J. A. Cross I. (New York, NY: Oxford University Press), 73–95.

[B18] Fitch W. T. (2013). Rhythmic cognition in humans and animals: distinguishing meter and pulse perception. Front. Syst. Neurosci. 7:68. 10.3389/fnsys.2013.00068 24198765 PMC3813894

[B19] Friberg A. Sundström A. (2002). Swing ratios and ensemble timing in jazz performance: evidence for a common rhythmic pattern. Music Percept. 19, 333–349. 10.1525/mp.2002.19.3.333

[B20] Gerischer C. (2003). O Suingue Baiano. Mikrorhythmische Phänomene in baianischer Perkussion. Frankfurt am Main: Peter Lang.

[B21] Gerischer C. (2006). Suingue baiano: rhythmic feeling and microrhythmic phenomena in brazilian percussion. Ethnomusicology 50, 99–119.

[B22] Goebl W. Palmer C. (2009). Synchronization of timing and motion among performing musicians. Music Percept. 26, 427–438. 10.1525/mp.2009.26.5.427

[B23] Goldberg D. (2015). Timing variations in two Balkan percussion performances. Empir. Musicol. Rev. 10, 305–328.

[B24] Grahn J. Brett M. (2007). Rhythm and beat perception in motor areas of the brain. Cogn. Neurosci. 19, 893–906. 10.1162/jocn.2007.19.5.893 17488212

[B25] Grube M. Cooper F. E. Chinnery P. F. Griffiths T. D. (2010). Dissociation of duration-based and beat-based auditory timing in cerebellar degeneration. Proc. Natl. Acad. Sci. U.S.A. 107, 11597–11601. 10.1073/pnas.0910473107 20534501 PMC2895141

[B26] Grube M. Griffiths T. D. (2009). Metricality-enhanced temporal encoding and the subjective perception of rhythmic sequences. Cortex 45, 72–79. 10.1016/j.cortex.2008.01.006 19058797

[B27] Hannon E. (2010). Musical enculturation: how young listeners construct musical knowledge through perceptual experience, in Neoconstructivism: The New Science of Cognitive Development, ed Johnson S. (Oxford: Oxford University Press), 132–156.

[B28] Hannon E. E. der Nederlanden V. B. Christina M. Tichko P. (2012b). Effects of perceptual experience on children's and adults' perception of unfamiliar rhythms. Ann. N.Y. Acad. Sci. 1252, 92–99. 10.1111/j.1749-6632.2012.06466.x 22524345

[B29] Hannon E. E. Soley G. Ullal S. (2012a). Familiarity overrides complexity in rhythm perception: a cross-cultural comparison of American and Turkish listeners. J. Exp. Psychol. 38, 543–548. 10.1037/a0027225 22352419

[B30] Hannon E. E. Trehub S. E. (2005a). Metrical categories in infancy and adulthood. Psychol. Sci. 16, 48–55. 10.1111/j.0956-7976.2005.00779.x 15660851

[B31] Hannon E. E. Trehub S. E. (2005b). Tuning in to musical rhythms: infants learn more readily than adults. Proc. Natl. Acad. Sci. U.S.A. 102, 12639–12643. 10.1073/pnas.0504254102 16105946 PMC1194930

[B32] Hasty C. (1997). Meter as Rhythm. Oxford: Oxford University Press.

[B33] Haugen M. R. (2014). Studying rhythmical structures in norwegian folk music and dance using motion capture technology: a case study of norwegian telespringar. Musikk og Tradisjon 28, 27–52.

[B34] Haugen M. R. Godøy R. I. (2014). Rhythmical structures in music and body movement in samba performance, in Proceedings of the ICMPC-APSCOM 2014 Joint Conference, ed Moo Kyoung Song (Yonsei University), 46–52. Available online at: http://vbn.aau.dk/files/204037394/icmpc_apscom_2014_Proceedings_.pdf

[B35] Henrich J. Ensminger J. McElreath R. Barr A. Barrett C. Bolyanatz A.. (2010b). Markets, religion, community size, and the evolution of fairness and punishment. Science 327, 1480–1484. 10.1126/science.1182238 20299588

[B36] Henrich J. Heine S. J. Norenzayan A. (2010a). The weirdest people in the world? Behav. Brain Sci. 33, 61–83. 10.1017/S0140525X0999152X 20550733

[B37] Holzapfel A. (2015). Relation between surface rhythm and rhythmic modes in Turkish makam music. J. Music Res. 44, 25–38. 10.1080/09298215.2014.939661

[B38] Jankowsky R. C. (2013). Rhythmic elasticity, metric ambiguity, and ritual teleology in tunisian stambeli. Anal. Approaches World Music 3.

[B39] Johansson M. (2009). Rhythm into Style. Studying Asymmetrical Grooves in Norwegian Folk Music. PhD thesis, Department of Musicology, University of Oslo.

[B40] Kalender B. Trehub S. E. Schellenberg E. G. (2013). Cross-cultural differences in meter perception. Psychol. Res. 77, 196–203. 10.1007/s00426-012-0427-y 22367155

[B41] Kubik G. (1988). Einige Grundbegriffe und -konzepte der afrikanischen Musikforschung, in Zum Verstehen Afrikanischer Musik, Kubik G. (Leipzig: Reclam), 52–113.

[B42] Kubik G. (1994). Theory of African Music, Vol. I. Wilhelmshaven: Florian Noetzel.

[B43] Kvifte T. (2007). Categories and timing. On the perception of meter. Ethnomusicology 51, 64–84.

[B44] Large E. W. (2008). Resonating to musical rhythm: theory and experiment, in Psychology of Time, ed Grondin S. (Bingley: Emerald Group Publishing), 189–232.

[B45] Large E. W. Gray P. M. (2015). Spontaneous tempo and rhythmic entrainment in a bonobo (*Pan paniscus*). J. Comp. Psychol. 129, 317–328. 10.1037/com0000011 26147705

[B46] Large E. W. Jones M. R. (1999). The dynamics of attending: how people track time-varying events. Psychol. Rev. 106, 119–159. 10.1037/0033-295X.106.1.119

[B47] Lerdahl F. Jackendoff R. (1983). A Generative Theory of Tonal Music. Cambridge: MIT Press.

[B48] London J. (2012). Hearing in Time: Psychological Aspects of Musical Meter, 2nd Edn. Oxford: Oxford University Press.

[B49] Longuet-Higgins H. C. Lee C. S. (1982). The perception of musical rhythms. Perception 11, 115–128. 10.1068/p110115 7155765

[B50] Longuet-Higgins H. C. Lee C. S. (1984). The rhythmic interpretation of monophonic music. Music Percept. 1, 424–441. 10.2307/40285271

[B51] Madison G. Merker B. (2002). On the limits of anisochrony in pulse attribution. Psychol. Res. 66, 201–207. 10.1007/s00426-001-0085-y 12192449

[B52] Marcus S. (2001). Rhythmic modes in middle-eastern music, in Garland Encyclopedia of World Music. The Near East, Vol. 6, eds Danielson V. Marcus S. Reynolds D. (New York, NY: Routledge), 89–92.

[B53] Marcus S. (2007). Music in Egypt: Experiencing Music, Expressing Culture. New York, NY: Oxford University Press.

[B54] McAuley J. D. Jones M. R. Holub S. Johnston H. M. Miller N. S. (2006). The time of our lives: life span development of timing and event tracking. J. Exp. Psychol. 135, 348–367. 10.1037/0096-3445.135.3.348 16846269

[B55] Merchant H. Grahn J. Trainor L. Rohrmeier M. Fitch W. T. (2015). Finding the beat: a neural perspective across humans and non-human primates. Philos. Trans. R. Soc. Lond. B. Biol. Sci. 370:20140093. 10.1098/rstb.2014.0093 25646516 PMC4321134

[B56] Merchant H. Honing H. (2014). Are non-human primates capable of rhythmic entrainment? Evidence for the gradual audiomotor evolution hypothesis. Front. Neurosci. 7:274. 10.3389/fnins.2013.00274 24478618 PMC3894452

[B57] Merker B. H. Madison G. S. Eckerdal P. (2009). On the role and origin of isochrony in human rhythmic entrainment. Cortex 45, 4–17. 10.1016/j.cortex.2008.06.011 19046745

[B58] Mirka D. (2009). Metric Manipulations in Haydn and Mozart: Chamber Music for Strings. New York, NY: Oxford University Press.

[B59] Moelants D. (2006). Perception and performance of aksak metres. Music. Sci. 10, 147–172. 10.1177/102986490601000201

[B60] Nozaradan S. (2014). Exploring how musical rhythm entrains brain activity with electroencephalogram frequency-tagging. Philos. Trans. R. Soc. Lond. B 369:20130393. 10.1098/rstb.2013.0393 25385771 PMC4240960

[B61] Nozaradan S. Peretz I. Mouraux A. (2012). Selective neuronal entrainment to the beat and meter embedded in a musical rhythm. J. Neurosci. 32, 17572–17581. 10.1523/jneurosci.3203-12.2012 23223281 PMC6621650

[B62] Nozaradan S. Zerouali Y. Peretz I. Mouraux A. (2015). Capturing with EEG the neural entrainment and coupling underlying sensorimotor synchronization to the beat. Cereb. Cortex 25, 736–747. 10.1093/cercor/bht261 24108804

[B63] Patel A. D. (2006). Musical rhythm, linguistic rhythm, and human evolution. Music Percept. 24, 99–104. 10.1525/mp.2006.24.1.99

[B64] Patel A. D. (2014). The evolutionary biology of musical rhythm: was darwin wrong? PLoS Biol. 12:e1001821. 10.1371/journal.pbio.1001821 24667562 PMC3965380

[B65] Patel A. D. Iversen J. R. Bregman M. R. Schulz I. (2009a). Studying synchronization to a musical beat in nonhuman animals. Ann. N.Y. Acad. Sci. 1169, 459–469. 10.1111/j.1749-6632.2009.04581.x 19673824

[B66] Patel A. D. Iversen J. R. Bregman M. R. Schulz I. (2009b). Experimental evidence for synchronization to a musical beat in a nonhuman animal. Curr. Biol. 19, 827–830. 10.1016/j.cub.2009.03.038 19409790

[B67] Patel A. D. Iversen J. R. Chen Y. Repp B. H. (2005). The influence of metricality and modality on synchronization with a beat. Exp. Brain Res. 163, 226–238. 10.1007/s00221-004-2159-8 15654589

[B68] Peretz I. (2006). The nature of music from a biological perspective. Cognition 100, 1–32. 10.1016/j.cognition.2005.11.004 16487953

[B69] Polak R. (2000). A musical instrument travels around the world: jenbe playing in Bamako, in West Africa, and Beyond. World Music 42, 7–46. 10.2307/41692764

[B70] Polak R. (2004). Festmusik als Arbeit, Trommeln als Beruf. Jenbe-Spieler in Einer Westafrikanischen Großstadt. Berlin: Reimer.

[B71] Polak R. (2005). Drumming for money and respect. the commercialization of traditional celebration music in Bamako, in Wari Matters: Ethnographic Explorations of Money in the Mande World, eds Jansen J. Wooten S. (Münster: LIT), 135–161.

[B72] Polak R. (2007). Performing audience: on the social constitution of focused interaction at celebrations in Mali. Anthropos 102, 3–18.

[B73] Polak R. (2010). Rhythmic feel as meter. non-isochronous beat subdivision in jembe music from Mali. Music Theory Online 16.

[B74] Polak R. (2012). Urban drumming: traditional celebration music in a West African City (Bamako), in Hip Hop Africa: New African Music in a Globalizing World, ed Charry E. (Bloomington, IN: Indiana University Press), 261–281.

[B75] Polak R. (2015). Pattern and variation in the timing of aksak meter: commentary on Goldberg. Empir. Musicol. Rev. 10, 329–340.

[B76] Polak R. London J. (2014). Timing and meter in mande drumming from Mali. Music Theory Online 20.

[B77] Povel D. J. Essens P. (1985). Perception of temporal patterns. Music Percept. 2, 411–440. 10.2307/40285311 3991313

[B78] Prögler J. A. (1995). Searching for swing: participatory discrepancies in the jazz rhythm section. Ethnomusicology 39, 21–54.

[B79] Rasch R. A. (1979). Synchronization in performed ensemble music. Acta Acustica United Acustica 43, 121–131.

[B80] Ravignani A. Bowling D. L. Fitch W. T. (2014). Chorusing, synchrony, and the evolutionary functions of rhythm. Front. Psychol. 5:1118. 10.3389/fpsyg.2014.01118 25346705 PMC4193405

[B81] Reinhard K. Stokes M. Reinhard U. (2015). Turkey, in Grove Music Online (Oxford Music Online: Oxford University Press). Available online at: http://www.oxfordmusiconline.com/subscriber/article/grove/music/44912

[B82] Repp B. H. (2003). Rate limits in sensorimotor synchronization with auditory and visual sequences: the synchronization threshold and the benefits and costs of interval subdivision. J. Mot. Behav. 35, 355–370. 10.1080/00222890309603156 14607773

[B83] Repp B. H. (2005). Sensorimotor synchronization: a review of the tapping literature. Psychon. Bull. Rev. 12, 969–992. 10.3758/BF03206433 16615317

[B84] Repp B. H. Su Y.-H. (2013). Sensorimotor synchronization: a review of recent research (2006–2012). Psychon. Bull. Rev. 20, 403–452. 10.3758/s13423-012-0371-2 23397235

[B85] Rose R. F. (1989). An Analysis of Timing in Jazz Rhythm Section Performance. Ph.D. dissertation, University of Texas at Austin.

[B86] Savage P. E. Brown S. Sakai E. Currie T. E. (2015). Statistical universals reveal the structures and functions of human music. Proc. Natl. Acad. Sci. U.S.A. 112, 8987–8992. 10.1073/pnas.1414495112 26124105 PMC4517223

[B87] Schachner A. Brady T. F. Pepperberg I. M. Hauser M. D. (2009). Spontaneous motor entrainment to music in multiple vocal mimicking species. Curr. Biol. 19, 831–836. 10.1016/j.cub.2009.03.061 19409786

[B88] Schulze H. H. (1989). Categorical perception of rhythmic patterns. Psychol. Res. 51, 10–15. 10.1007/bf00309270

[B89] Shaffer L. H. (1984). Timing in solo and duet piano performances. Q. J. Exp. Psychol. 36, 577–595. 10.1080/14640748408402180

[B90] Snyder J. S. Large E. W. (2005). Gamma-band activity reflects the metric structure of rhythmic tone sequences. Cogn. Brain Res. 24, 117–126. 10.1016/j.cogbrainres.2004.12.014 15922164

[B91] Tenzer M. (2011). Generalized representations of musical time and periodic structures. Ethnomusicology 55, 369–386. 10.5406/ethnomusicology.55.3.0369

[B92] Timmers R. Endo S. Bradbury A. Wing A. M. (2014). Synchronization and leadership in string quartet performance: a case study of auditory and visual cues. Front. Psychol. 5:645. 10.3389/fpsyg.2014.00645 25002856 PMC4066619

[B93] Ullal-Gupta S. Hannon E. E. Snyder J. S. (2014). Tapping to a slow tempo in the presence of simple and complex meters reveals experience-specific biases for processing music. PLoS ONE 9:e102962. 10.1371/journal.pone.0102962 25075514 PMC4116158

[B94] Waterman R. (1952). African Influence on the Music of the Americas, in Acculturation in the Americas, ed Tax S. (Chicago, IL: University of Chicago Press), 207–218.

[B95] Wing A. M. Kristofferson A. B. (1973). Response delays and the timing of discrete motor responses. Percept. Psychophys. 14, 5–12. 10.3758/BF03198607

